# Henry De Mondeville, the Man and His Writings

**Published:** 1903-03

**Authors:** Charles Greene Cumston

**Affiliations:** Boston, Mass.


### BUFFALO MEDICAL JOURNAL.

VOL. XLII.—LVIII. MARCH, 1903. No. 8.

### ORIGINAL COMMUNICATIONS.

### Henry de Mondeville, the Man and His Writings.

**With Translation of Several Chapters of his Works.**

By CHARLES GREENE CUMSTON, M. D., Boston, Mass.

*(Continued from February Number.)*

CHAPTER III.

RESOLUTIVA AND THE MANNER OF USING THEM.

Like the previous chapter this one is also divided into three divisions:

The first part has two subdivisions:

(1) Avicenna in Canon II., Div. I., Chapter 4, and Serapio in his Aggregetiones, sermon 4, de divisione duarum medicinarum, both say: The resolutiva distribute humors, convert them into vapor and gradually draw them from the depths of the tissues, until by their continued application all humor has been withdrawn. They must be dry, distributing, opening and not drying; hot, in order that they may draw the distributed humor from the member; distributive, in order that they may distribute the substance of the humor which we seek to dissolve; opening in order that they may open the pores of the skin sufficiently and can dilate them so that the dissolved materia may make its exit; nonciccative, so that they do not absorb the moisture by their dispelling and drying qualities, which softens the entire materia which is to be resorbed. In the chapters of Avicenna and Serapio, it will be seen what constitutes a warming, distributive, opening and drying medicine.

(2) When treating an abscess we must, shall and can, if proceeding correctly, apply resolutiva in two cases: firstly, when we do not dare to repercuss, as in this case, we are confronted with one of the nineteen cases mentioned in another rule of the chapter on generalities; secondly, when we try to repercuss and are unsuccessful, because the body is full and cannot absorb anything or because the materia does not obey. Here, also, resolutiva are contraindicated (provided, of course, that the abscess is produced by some internal cause), unless before a proper purge has been administered. This we have already demonstrated in the chapter dealing with the general treatment of abscess. The resolutiva agree with the maturativa in two points, and they deviate from them in one point, at least for the time being. Firstly, they resemble each other in that they relieve pain; and, secondly, in that they sometimes can be substituted one for the other; for example, when a resolutivum is applied to such a large quantity of materia that the pores are inadequate for the exit of the matter, then the resolutivum has a maturing effect at the same time. If, on the other hand, a maturing remedy is applied to a subtle quantity of matter, it dissolves it. Thus, it frequently happens that one and the same remedy, like the plaster of common juice, sometimes matures, sometimes loosens up the process. The difference between these remedies consists in that the resolutiva will produce an opening in hot materia, and the maturativa with their hot quality will block up. From what has been said two facts are obtained: (1) the resolutivum is capable of dissolving the subtlety of any materia, and densifies its residuum; (2) it is capable of dissolving the subtlety in the abscess and of maturing the dense part.

II. The resolutiva are either simple or compound.

The simple ones are as follows: camomile, which alone is of any value, since it does not attract more than it dissolves, honey clover, paritaria, the white or forest malva, fumatory, anetum, cabbage, the nettle, enula, borrage, buglossa, sambucus, ebulus, valerian, the seeds of cabbage, anetum, nettle, malva, parsley, apium, fennel, furfur hordei, fabæ, (fabarum?), cicer (?), orobus, (bran of barley, beans, peas, forest pea), thick bread crumbs, the fat of the goose, duck, fowl and pig, all kinds of marrow, mastix, incense, myrrhs, gum ammoniac, separium, galbanum, apoponax, and all kinds of fine gum, lapdanum, ysopus humida, turpentine, wax, the sediment of wax, butter and similar substances.

The composite resolutiva made from the above and similar drugs are oils, unguentums, plasters, poultices, pastes and ferments.

There are six oils: (1) a mixture of equal parts of anetum oil and the common ripe oil; (2) oil of camomile (from the blossoms), with common ripe oil; (3) oil of costos root, composed as follows: rp. costi unc., 1; pepper, pyrethri, euphorbii ana unc., 1-3; castor oil unc., 1, must be pounded together, passed through a sieve and dissolved in ½ pound of either lily or spicanard oil; it dissolves the cold humor and tensifies the nerves; (4) oil of the large camomile: rp. freshly dried camomile blossoms, buckthorn, linseed ana unc., 2, are placed in a glass jar containing 20 ounces of ripe oil. The oil may be allowed to stand in the sun or near a fire, in a well, or in a pot placed under the earth; it dissolves, warms, and relieves cold pain; (5) oil of lily, compounded as follows: rp. oil unc., 2; lily blossoms, without the lower saffron yellow colored parts, No. 30, cassia, radix costi, mastix, balm of Mecca, saffron, ana unc., 1; cloves, cinnamon, ana unc., ½, are all pounded together and placed in a glass jar in a shady place, excepting the lilies which are put separately in a little sack and which is hung into the mixture and then removed after a month, so that they shall not spoil and thus spoil all the oil. When warmed this oil warms, stops the cold pain, especially of the kidneys and uterus, and has no irritating effect; (6) oil of mastix: rp. moderately ripe olive oil unc., 3; mastix drachm, 6; to be boiled in a very large receptacle until the mastix is dissolved. This remedy has a marked dissolving effect and is excellent in cases of abscess in the neighborhood of the stomach, liver or spleen, relieves the pain and prevents fits, especially arising from the cold materia.

Ointments can be made from any one of the above mentioned oils by mixing them with the aforesaid powders and with wax, observing the quantities alluded to. We will here give five of them: (1) rp. oil of camomile unc., 3; wax unc., ½; buck's horn in powder, linseed ana unc., ½; are boiled and passed through a sieve; this dissolves and matures without attraction; (2) rp. oil of lilies unc., 3; wax unc., ½; seed of malva ana unc., ½, are mixed—dissolves like the preceding formula and matures hot materiæ; (3) rp. bdellium, sagapegum ana unc., ½; turpentine unc., 2, dissolve in vinegar until a saturated solution is obtained, then mix with turpentine, after a solution is obtained add two ounces of oil of lilies and pass through a sieve—absorbs cold abscesses; (4) rp. oil of camomile or anetum, drachms 6; wax, drachms 2; fat of the duck and fowl ana, drachms 2; seed of anetum and camomile flowers ana, drachms 2,—the same process as above. (5) This is neither an ointment, properly speaking, nor a plaster, but is between the two and is termed moist ysop. Rp. fat wool, such as is found between the ribs and mammæ of sheep, over this rain water in desired amount is poured, so that it completely covers the wool and is allowed to stand twenty-four hours. It is then boiled over a moderate fire, cooled and passed through a sieve, then it is again boiled over a moderate fire in a metal vessel, being stirred with a wooden spoon until it becomes thick. This absorbs and softens like an ointment and can be used like an oil for making resolutive salves, plasters and any kind of resolutive preparations.

We have three resolutive plasters: (1) the diachylon plaster of Rhazes: rp. ripe oil unc., 5; litharge finely powdered unc., 1; buck's horn and linseed ana unc., 2; marshmallow unc., 1. Same preparation as given before. But if it is to be employed for dissolving scrofula, 1 ounce of dried powdered violet root should be added; but it will not dissolve scrofulous cysts and similar things; carbuncles are matured with the addition of the violet root; (2) lead plaster, according to Mesue: rp. pounded and sifted litharge unc., 12; oil of camomile, oil of anetum, and oil of violet root ana unc., 8; mucilage of marshmallow, powdered buck's horn, linseed, dried greasy figs, stoned grapes, juice of the violet root and squill, moist ysop, fish glue ana unc., 12; turpentine unc., 3; white resin, yellow wax ana unc., 2. These are boiled until a consistency between ointment and plaster is obtained. This ointment dissolves cold materia and softens a hard one; (3) common lead plaster, as described in the Antidotarius of Nicolaus: rp. old oil unc., 4; spumæ argenti unc., 36; malva and marshmallow root, buck's horn, linseed ana unc., 12, until a mucilaginous decoction is obtained, put one ounce on plaster. Regarding the manner of compounding this, we have already spoken of it in the introduction to this Antidotarium. The plaster is used for boils and for dissolution of small hot abscesses, when it is indicated to dissolve the matter. In the other case it digests, draws out, cleanses, regenerates, heals and has a good effect in curing from the beginning. In large hot abscesses its effect is favorable if in spite of the opening (incision?) they do not become healthy, and where the apostematic dyscrasia still remains. It is also indicated in cold abscess; it must therefore be removed twice daily and dried by means of wiping; after being dried it is freshened up by friction with the thumb and then the ointment is replaced. It has a similar efficaciousness in pains and chronic swellings of the joints, and in pains of the intestine; dissolved in oil of mastix and placed on cut nerves, which are contracted by plethora; it is also applied to wounds of bad blooded patients.

We have four poultices: (1) rp. camomile flowers, seed of anetum unc., 2; powdered buck's horn, linseed and barley seed ana unc., 8; oil of camomile and anetum ana unc., 1, are to be boiled in a sufficient quantity of water and then pounded together and applied after repercussion and discharges. It absorbs abscesses and hot humors, and prepares hot abscesses for maturing; (2) rp. forest malva, cabbage leaves, camomile flowers ana, part 1, or one handful of each are boiled with the water and pounded in boiled water, and then add anetum or cabbage seed ana, part 1, powdered sulphur, parts 2; (3) rp. fennel, anis seed, anetum seed ana unc., 2; lupine, powdered buck's horn and linseed ana unc., 3; oil of lilies unc., 1, to be boiled in water, then pounded and afterwards add a little oil or vinegar; (4) our poultice or plaster of malva seed, as already described in Chapter II., Doctrine 2, Tract 2, in the seventh section of said chapter, which treats of the prevention and treatment of hot abscess, in the fifth introductory Notabel, in which we have also discussed its numerous well-confirmed excellent properties.

Paste poultices which the older surgeons used as resolutiva of hot wounds appear to me rather more relieving remedies in cases which call for suppuration (and the ancients liked these especially), are made of four parts of water to which is added a sufficient quantity of flour. After this is mixed, one part oil is added and then the whole is boiled to the consistency of a soft paste and applied lukewarm.

Fomentations are made from the decoctions of the above simple remedies. They must always be applied immediately before the application of the above mentioned compound local remedies.

III. Explanations, of which there are four: (1) if simple medicines are boiled for resolution, one part of the hot decoction is to be laid aside and therewith poultice the affected part until it begins to redden and swell, then the proper local remedies are at once applied; (2) if during the process of resolution the subtle (matter) absorbs itself from materia and if the residuum hardens, then maturing remedies are combined with the loosening ones, and they are alternatively applied until the desired condition is obtained. Maturing remedies, as much as is necessary, are considered later (3) if in the process of resolution all is not loosened, what one proposes to have loosened, and if the materia on the contrary, appears to be thick and gluey, therewith appearing to proceed towards maturing, then the maturing should be favored by local remedies which we have yet to consider, because the surgeon must always imitate nature, which later will always correctly operate; (4) the local resolutiva, maturativa, and mundificativa must not be of hard consistency, so that they will not injure the affected parts by their harshness, and will not, owing to the violence of the pain, attract the humors from some other part.

As these two chapters are taken from de Mondeville's treatise on drugs used in the treatment of wounds and abscesses, I think it will not be without interest to consider somewhat at length the rules he has laid down for the treatment of the latter process, which was called in those days an apostasis and simply means an accumulation of pus. The physicians of antiquity and the middles ages did not include in this conception an accumulation of pus as such, but, as is evident from the etymology, it means in a wider sense the conception of extension, an increase in height, that is, rising above the level from the surface of the body. A swelling, which under certain conditions, especially resulting from an external or internal inflammatory process, they called apostema.

The ancient writers assumed that the "*vix medicatrix naturæ*" endeavors to provide an outlet for the so-called "*materia peccans*" contained within the body and by this means endeavored to eliminate it. The formation of pus was in their way of thinking a matter of secondary consideration only, while, on the other hand, the therapeutical measures employed by them show that they were not convinced of the absolute necessity of the formation of pus.

Various repelling remedies, the so-called repercussiva, to which I have already alluded in the translation above given, were employed by the older surgeons for the purpose of preventing the formation of pus.

The works of Hippocrates refer in innumerable places to the words apostasis and apostema, usually in the sense in which at the present time the word apostate is used in its primary sense, that is, as a transition or a termination of one disease into another. "Οἱ δὲ διαφεύγοντες φθάνουσι μετ' ἀποστήματος" qui vero effugiunt aut cum abscessu aut . . . effugiunt. (Those who recover in certain severe epidemic diseases, or from severe fevers, are cured by the formation of an apostema.) Thus, for example, it is stated in the twelfth chapter of the well known treatise on the manner of living in acute diseases, by the immortal father of medicine.

In the expression εἰς ἄρθρα ἀφίστασθαι, which literally means "to throw itself on the articulations," is to be found in the works of Hippocrates, the original meaning I take it is as a critical phenomenon in general, and this meaning was attributed to the word as is most plainly shown if the text is carefully read. Each termination of a disease, no matter whether δι' ἔκκρισιν (by elimination), or κατ' ἀπόθεσιν (by deposit of sediment), is called apostasis, as has been pointed out by Galen in his well known commentary to the sixth book on epidemics by Hippocrates. In another part of his commentaries on the epidemics of Hippocrates, Galen confirms that the physician of Cos designated the transitions, the so-called metastases, as "ἀποστάσεις". According to him they could arise either by the veins, arteries, the abdomen, in the skin, bones, the medulla, or to other "ἐκροάς," that is to say, outlets, to the mouth, ears, nose and genital organs including the uterus.

Galen derives the word apostema directly from "ἀφίστημι" in his two books on Therapeutics which were dedicated to Glaukon, and explains the designation particularly: "ὅτι ἀλλήλων ἀφίσταται τὰ πρότερον ἀλλήλων ψαύοντα," because by the formation of the apostema that which formerly touched had now become separated.

In the works of Paulus, of Aegina, the renowned physician of the Byzantinian period, whose principal work attained such a large reputation, especially for surgery, the word apostasis is strictly confined to the process of suppuration. At the commencement of the 65th chapter of the 3rd book at which abscess of the vulva is discussed, the following passage occurs: "τῆς φλεγμονῆς εἰς ἀπόστασιν μεταβαλλομένης, etc., that is to say, when inflammation transforms into suppuration, the symptoms described increase in intensity while irregular elevations of the temperature accompanied with chills, set in.[1]

It is not to be a matter of surprise that in the later authors

1. For these explanations I am indebted to the monograph of Dr. Max Neuhaus, Berlin, 1897.

of the middle ages the expressions apostema and abscessus are used merely in the wider meaning of "exitura," meaning simply the termination of an affection with a tumor-like formation on the surface of the body. This is especially evident in the writings of Avicenna, who as is well known has supplied us with a clue for the understanding of the medieval medical literature. One of the passages in the famous Canon is as follows: "Eminentiæ enim sunt apostemata parua, sicut apostemata sunt eminentiæ magnæ. Et in apostemate enim omnia ægritudinum genera reperiuntur. In eo namque reperitur ægritudo malitiæ complexionis . . . . . Et invenitur in ipso ægritudo communis, quæ est continuitatis solutio. Nullum enim accidit apostema nisi procul dubio continuitas soluatur; propterea, quod effunduntur materiae superfluæ ad membrum aposteatum et inter partes penetrant ipsius, et separationem inter unas faciunt et alias, etc." So speaks Avicenna, and how important the doctrine of abscess formation and the treatment of abscess in general was to the medieval surgeon, is most plainly evident from the fact that Guy de Chauliac in his Chiururgia magna places his chapter on abscess immediately after his treatise of anatomy. The following is a free translation into English of what de Mondeville has written on abscess, and in what I have here given the reader will be able to glean an insight into the conception that the medieval physicians had formed of abscess formation, and especially on the general principles for their treatment. Taking it all together, de Mondeville has given us a résumé of the teachings of his predecessors commencing with Avicenna and the representatives of School of Salerno, and this section of de Mondeville's treatise is an excellent basis for the formation of a historical appreciation of the work, because later surgeons as, for example, de Chauliac, make no additions or innovations on this subject, and we here insert the English version of his teachings.

### Chapter II. Doctrine II. Treatise III.

On the ordinary treatment of abscess, in which will not be discussed the special forms of abscess, or the materia from which they originate, excepting when quoting examples.

We have three general points to be considered: (1) the recognition, (2) the treatment, (3) the explanation.

1. *Definition.*—An abscess is a swelling or thickening in a member when this increases its natural size, although barber surgeons and laymen, who are all ignorant, do not believe that this is an abscess when there is no suppuration, or where no suppuration appears to come of necessity, and when the swelling is not great. This, however, is in contradiction to Avicenna (Chapter II., on Complicated Diseases), who says that small protrusions are small abscesses, while large ones are large abscesses.

*Division*—Abscesses originate from the humors of the body, from the water and from the gas. Abscesses arising from the fluids of the body originate in the blood or bile, in the mucus, or the atribile. These humoral abscesses may also originate from an over abundance of bad qualities of the natural humors. Consequently, abscesses originating from natural or unnatural humors may be composed of a single humor without the admixture of another, as, for example, pure blood. Or, they may be composed of several humors mixed together, as, for example, blood with bile or mucus. They may also be produced by certain internal causes, such as an abundance or bad condition of the humors, or by some external cause, such as a fall or a blow, or by both causes combined. In other cases the abscess is due to an occlusion, others by derivation, while still others arise from a combination of both these causes. Some project considerably, others not at all. Some protrude in part, while the remainder does not. They may originate in very important organs, in their neighborhood or at some distance from them. Then again, in the fleshy parts, in a body in the best of health or in one in bad condition, in strong bodies as well as in delicate ones.

*Causes.*—In order not to lose one's self in the intricacies of the unknown it is necessary to learn how each abscess has originated, whether by obstruction, derivation or in any other manner. Thus it may be said that an abscess originating at any part of the body on account of an excessive alimentation of that part, inasmuch as for some reason the nutritive substances cannot be transformed into the tissues of that part, has originated from an obstruction, that is to say, an accumulation, and in no other manner. The causes of obstruction are an excess or bad condition of the nourishment flowing to the part, and a weakness of the digestive or expelling power of the member itself. An abscess in any part of the body which does not thus originate in this fashion, but arises from substances which have been transferred to it from some other member of the body, and to which said member cannot resist for one or several reasons, is said to have originated by derivation, that is to say, by deposit.

Adjuvant causes are weakness in the receiving member, strength of the repelling member, deeper location of one or the other, the quantity or bad condition of the transmitted substances, the caliber of the veins leading to the receiving part, or small caliber of the veins leading from it, patentcy of the receiving parts and of the glands.

There are still other causes, all of which coöperate in the formation of abscesses, but which are not necessary to enumerate here, as they have already been considered when speaking of the cause of ulcers, in Chapter I., Doctrine II., Treatise II. The recognition of abscess is greatly helped by the knowledge of their manner of originating, as will be shown in the Explanations.

*Symptoms.*—Regarding the symptoms of abscess in general, Avicenna says that the senses, that is to say, both sight and touch, demonstrate those abscesses that are visible, and he immediately adds that it is very difficult to enumerate all the signs of abscesses, and even were it as easy it would require lengthy explanations; therefore, it is better that the discussion of these symptoms should be deferred until the question of special abscesses is taken up. As an additional reason for this we may say that since abscesses in general do not offer any symptoms but those found in individual cases, as in phlegmon, erysipelas, and the like, the symptoms of abscesses in general in no way differ from what will be shown in the specific cases which are to follow. From what has been said certain positive symptoms may be gleaned by means of which, when we meet with an abscess, it can be diagnosticated by the experienced eye and touch.

The above mentioned definition in its single parts, the division with its variations, the causes enumerated, as well as innumerable others, along with the symptoms referred to to which others should be added, must be observed by the physician if he desires to successfully treat abscesses, because whether any specific condition, or two or more, are found present in a given case of abscess, or if they are wanting, it is necessary to proceed in some way or other, and this is characteristic for the entire general doctrine of abscesses and similar affections.

There are three kinds of general treatment—namely: (1) the preventive, (2) the curative, (3) the paliative. The preventive treatment, both in its application and indications has already been alluded to in the preface to Doctrine II., Treatise II. Prophylaxis is a triple one according to the various periods of the disease to which it is applied: (1) the treatment of an abscess that is not yet formed, but which will be if not prevented from so doing; (2) the prevention where an abscess already exists, and will become larger if not prevented, and (3) the prevention in case a large abscess should suppurate.

The first of these is effected by four factors—namely, by the necessary control of the six unnatural things (aer, esca, quies, repletio, gaudia, somnus), by abstinence, such as the experienced physician knows how to prescribe against the cause of the disease, by medical and surgical evacuation carried out at the proper time and place.

The doctrine and prescribing of these evacuations are given in Doctrine I., Section 2, in the fifth part of the principal Chapter I., which deals with the manner of evacuating and the potions for the wounded, and also in the Explanations of the section referred to; and also by protection against contusions and similar external injuries. But this preventive treatment must not be obtained by evacuations brought about by derivation, because it is impossible to know from which spot they are to be diverted. For there is no doubt that if a body is kept in a proper condition of health no abscess will ever arise in it.

Prevention is obtained by means of three things, namely—a proper diet and evacuation, which is spoken of in Doctrine I., and if all is not said about it there, it remains for the physician to supplement it; and it may be that a local treatment will produce certain results aside from those obtained by the special ones.

Local treatments are of three different kinds: protective measures of the diseased spot, repercussion above the abscess (only in places and cases where repercussion can be borne by the patient, such cases being described in the chapters treating this matter); the application to the abscess of dissolving remedies, which are sometimes applied alone and in others in conjunction with repercussiva.

Thirdly, preventive treatment is effected in the case of a large or fully developed abscess, in order that it may not suppurate by three things—namely, good living, evacuations as above indicated, local dissolvent, and other measures, as will be described later when considering special cases. The various local treatments will be discussed in the Doctrine devoted to antidotes.

Galen formulates rules regarding that prevention in the 10th Book of the Megategni Chapter, which begins "nunc auten conderdentum, etc.," as well as Hali in his commentaries on the Microtegni, where he calls the preventive cause the preventive treatment to which the surgeon may have recourse.

In regard to the curative treatment there are eighteen general rules:

1. Each abscess arising from an internal cause has its source in a superabundance of or a decomposition of humors, or in gas or water or in both, or in several other things mentioned. In abscess arising from an external cause they are sometimes occasioned by and sometimes without those causes.

2. Every abscess is either repelled, hardened, or suppurates, and then follows the termination of which Avicenna says in speaking of acute abscess, that no abscess may be considered as nearing its end so long as pus is formed in it.

3. If an abscess can be healed by an operation without causing suppuration, it should be done, because in all cases where pus forms in large quantities fever and other dangers naturally ensue.

4. The condition of the abscess should be carefully observed by the surgeon, for his aim should be to bring about a cure without suppuration, whether the abscess is derived from external or internal causes, so that if an internal cause be connected with an excessive amount of humor and great danger involved, the treatment should begin with an evacuation. If, on the other hand, decomposed humors are present, and if the abscess be small, a rectification is sufficient. Not every unfavorable condition of the entire economy can be cured by its contrary, that is, by evacuation, for occasionally a restrictive diet is sufficient where there is plenitude, which means that the patient is fleshy and his organism in bad condition. Care must be taken not to apply local remedies in those cases where an evacuation is indicated before this can be done with propriety, because this repercussivum would not repel, and therefore would have no action on the matter. For this reason Galen says: "If we wish to collect that which is bubbling, it would not be absorbed by the full body; it happens on the contrary that if repercussiva are applied, even if for a time pain should be relieved, they nevertheless harden the abscess so that in the end the patient will be a greater sufferer." The same mistake is made in applying dissolvent remedies, because, according to Galen, with the exception of chamomile, every dissolvent attracts more than it dissolves. He also says in II. Metategni, Chapter III., "si auten e contrario, etc." Each dissolvent is warm, everything warm attracts except when moderate.

In a like manner if an irritative is applied it will at some time or other attract fine matter by means of its warmth, which is already on the point of liquidating, and will also increase the pain and produce an enormous abscess. With regard to purgatives, something has already been said in Chapter III., Doctrine I., Treatise II. The remainder, which is sufficient for the experienced surgeon, will be found in Doctrine III.

5. An abscess of large size occurring in a full habit is treated by the four following rules, namely: (1) general purging; (2) local purging; (3) local repercussion and local protection; (4) by solution when repelling is not sufficient. This Galen says, in the chapter above quoted, when he speaks both as physician and surgeon, adding: "If what we have mentioned is not sufficient, one must resort to ripening, but this holds good immediately after the first rules given, as is explained in the above mentioned second rule—namely, that the ripened abscess should be opened."

6. In the case of a plethoric patient no severe evacuation should be given, nor should any incision be made in the diseased part excepting to give exit to the pus, because one should not evacuate, since owing to the pain of evacuation more matter will be drawn to the spot (according to Galen); therefore it results that if an abscess forms in the anus no evacuation should be allowed by means of laxatives in a plethoric patient, but should be obtained by vomiting, and if an abscess occurs over the umbilicus, the evacuation should be produced by laxatives. It thus results that under no circumstances should hot local remedies be applied, because they attract more matter from other parts. Averrhoes, however, corrects this rule in the 7th Book of his "Colliget" as follows: "When the principal bulk of an abscess has been compensated, evacuation may be made through the affected part.

7. When an abscess is commencing in a plethoric patient, the surgeon should generally begin treatment by means of a derivation, according to the rules of medicine, after purging has been produced, and in the commencement in a nonplethoric patient where no purgative has been used, by means of repercussiva, excepting in those cases where either before or after the purging, even in a nonplethoric patient, a repercussive remedy is not indicated: (*a*) where the matter is thick or solid, so that the surgeon cannot repel it even where he should and ought to do so, because the matter is not suitable for this and on the contrary becomes more solid when an attempt is made to repel it; (*b*) those cases where the matter is cold, because it would not obey; (*c*) if it can be done by blocking it off, because it is better that such matter should be discharged by the member in which it is collected than by another, which nature always ascertains; (*d*) where there is a superabundance of matter, because in this case it cannot be repelled; (*e*) in a pregnant woman, because it is to be feared that the repercussivum might injure the fetus; (*f*) when a vital part is affected, because it is better for the matter to be drawn out through a nonvital part; (*g*) if suppuration is deeply seated, as for example, in the hip; (*h*) if the patient has a critical abscess, because it might kill him; (*i*) when the patient is on the way to recovery, because the pus of such an abscess represents the remainder of the former disease, which the organism expels because it is strengthened and its functions are normally carried out; (*j*) if it is in an emunctorium, because, as has been shown by all writers, no repelling but an energetic expelling method is indicated, and if necessary by gas producing means, as will be shown later; (*k*) in a youthful individual, because owing to the weak condition no repulsion will take place; (*l*) in an aged person, for the same reason; (*m*) in the neighborhood of a vital part, because if it is repelled some danger might be created for the part involved; (*n*) if the matter be hard, because it cannot be repelled and would become still more dense; (*o*) if the matter be of a virulent kind, because there will then be danger that it would be carried to the vital parts; (*p*) if the matter be poisonous, for the same reason; (*q*) if the pus is deposited in a joint, because in this case the repelled matter might enter the cavity of the joint, destroying the nerves and ligaments; (*r*) if the abscess is near the anus, because repelling causes it to be fistulous; (*s*) if it has been produced by some external cause, because it is better that the decomposed pus should be withdrawn through the skin of the injured part than that it should escape inwardly.

These nineteen rules are laid down in the well known Salernitanian rule: "Thickness, cold, obstruction, fulness, pregnancy, importance of the part of the body, subsequent crisis, convalescence, secretion, youth, age, neighborhood of a vital part, density, poison, seated in the joints and anus, external causes. In these cases thou shalt not repel neither prior to nor after purging, but in the other cases thou shalt repel after thou hast purged the full body, or not have purged the weakened body."

8. An abscess due to an external cause in a well preserved body does not require to be opened nor to have local protection; on the contrary it is sufficient to repel and strengthen the part by means of local remedies. Avicenna says, however, regarding the treatment of abscess, that dissolvent and softening remedies are to be applied in the beginning, but if the habit be plethoric an evacuation should precede and also other measures are to precede, which will be mentioned further on.

9. In cases of acute abscess cold applications should be made after purgation, because contrasts are cured by contrasts. Thus Galen says: "Therefore the repercussiva for acute abscess are pure styptica, dry and cold remedies, such as chimolea and similar ones, and the repercussiva for cold abscesses, if I may term them such, are to be composed of this and other warm subtle dissolving remedies, or simple remedies which are both styptic and dissolving in their quality, such as absinth and schoeanthus." Thus also does Avicenna speak on the treatment of abscesses.

10. A part of the seat of an abscess must not be moved violently nor should it be allowed to hang down. Thus if the foot be the seat of the trouble, the patient should neither walk nor stand. If the hand is involved, it should be suspended from the neck and not allowed to hang down. (Galen, de ingenio, V., Chap. IV.)

11. A member which has solid pus deeply seated, or which is covered by a thick skin, or both, requires stronger remedies than where the opposite conditions prevail, as for instance, in the foot or nipple.

12. An evacuation should never be made below the abscess, that is, so that the abscess is situated between the chest and the part through which the exit is made. Thus, in the case of an abscess of the tibia, the evacuation must not be effected through the foot, no matter what may be the stage of the disease, be it that the humors of the abscess are really discharged by means of the said evacution; be it that such is not the case, from the entire body or from the member involved, or from both, several humors will be withdrawn which otherwise would follow the current of the evacuation towards the abscess, and these will remain in the abscess after evacuation has ceased, will enlarge it and will in the course of time become decomposed by remaining within it.

13. If an abscess is in its early stage or is growing, or if it is seated in a member very remote from the chest, such as the hand or foot, the opposite member must be violently used or it must be tied and balasted by a stone. Thus speaks Avicenna, Chap. XXV., on the treatment of abscess. For in this manner the abscess is compensated or destroyed, or at least reduced in size, because by means of such methods the flowing humor is withdrawn from a commencing abscess.

14. If an abscess commences in an emunctory, the matter should be drawn towards it thoroughly, by means of dry cupping if not possible with other means.

15. If an abscess whose complete maturity has been awaited becomes completely ripe, the symptoms of this being a decrease of the fever, redness, pulsation and pain, improvement in sleep, etc., then if it does not spontaneously open it should be incised with the knife, but observing the rules and cautions which have or are to be mentioned, and also taking into account the condition of the abscess, the quantity and nature of the matter, as well as the localisation of the abscess, for instance, near the anus or the head, etc., as well as observing the patient's condition, that is to say, whether he is a hardy peasant, or a weak inhabitant of a city, whether he is an old man or a boy, whether he is strong or weak; and also the influences of the heavens, for instance, whether the moon be free in the sky or covered, whether the moon is on the decline or has gone down, whether it is not at the end of the scales or in the beginning of the scorpion, or not in any sign, which designates the number that is to be operated on. Such signs can be recognised by the astronomer and can also be found in the planet almanac, or in the small astronomical treatise entitled "Circa instans."

The incision must be made for two reasons: firstly, because pus burrows too much; and secondly, because if we await a spontaneous opening a circular ulcer results, which heals in some cases with difficulty, while when an incision is made a longitudinal ulcer is created.

16. In cases of abscesses which have already opened, no softening or moistening remedies are to be applied, but drying ones should be used which are appropriate for the ulcer, because we should now be more preoccupied for the cure of the ulcer than for the abscess, because the former has been added, and is more important than the latter lesion.

17. No abscess should be opened before it has sufficiently ripened excepting in six cases. The symptoms of ripening are various, according to the variety of the pus, as will be seen in the special cases. The reason for this rule is that a ripe part, which is extracted, helps another in maturation; another reason is that if an unripe abscess is opened the pain is more violent, and pain destroys the power of resistance and increases the supply of humors. The first of the six cases to be excepted is the following: if the pus is about to damage the member, or has undergone coction, because then it is better to open an unripe abscess than to lose the limb; secondly, if the abscess is situated near some vital part, in order that the matter which is retained too long in the abscess may not be drawn towards this vital part; thirdly, if it be situated in an emunctory, for the same reason; fourthly, if it be in the joints, because the pus might spread and destroy the ligaments; fifthly, at the anus, because the discharging fecal channels easily become fistulous; sixthly, if the abscess arises from a thick heavy mucus wherever it be, because occasionally such an abscess is filled with liquefied fleshy matter before it becomes ripe.

18. As soon as the abscess is ripe it should be immediately opened so that the pus may be evacuated, observing those rules and other indications which are to be observed, excepting in three cases. The reason for that rule is to be found in Galen; all things which are in the body contranaturam must be extracted, etc., and this is still more clearly demonstrated from the declarations which precede that part, and which are contained in Chapter I., Doctrine 1, Tractus 2, on the Treatment of Wounds. The first exception is where an abscess is composed of humor having undergone coction, as anthrax, etc., because the pus is as thick and as tough as skin and sinews, and because the pus will not escape after an incision is made. In this case, too, the strength of the patient is quite exhausted, and if the operation be made and the patient should die, the surgeon is responsible for his death. The third instance is where the surgeon requires money, but has as yet not received it, according to the principle: take the money while the disease still hurts, that is, because if the abscess is opened and if the fever and pain subsides, the silly surgeon frequently has to go without his fee.

(*Concluded in the April number.*)

